# Supplementary material for: Novel Driver Strength Index highlights important cancer genes in TCGA PanCanAtlas patients
Source: PeerJ. 2022 Aug 11;10:e13860. doi: 10.7717/peerj.13860 (PMC9375969; doi:10.7717/peerj.13860)
Supplement: Supplemental Information 2 [file peerj-10-13860-s002.zip › COHORTS/genes plots/detailed/2021_8_16_14_9_distribution_events_detailed_21.pdf]

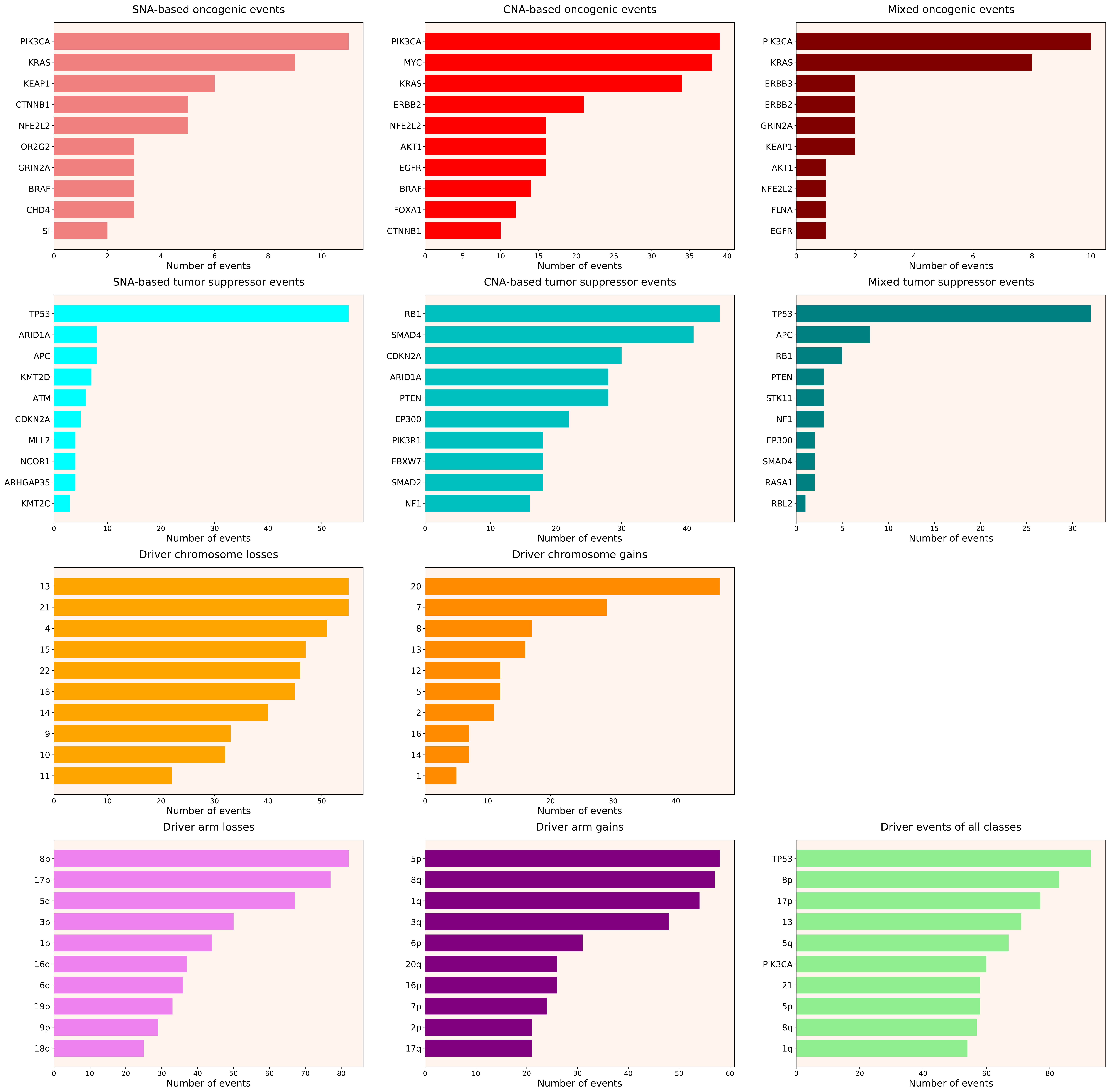
SNA-based oncogenic events
PIK3CA
KRAS
KEAP1
CTNNB1
NFE2L2
OR2G2
GRIN2A
BRAF
CHD4
SI
Number of events
CNA-based oncogenic events
PIK3CA
MYC
KRAS
ERBB2
NFE2L2
AKT1
EGFR
BRAF
FOXA1
CTNNB1
Number of events
Mixed oncogenic events
PIK3CA
KRAS
ERBB3
ERBB2
GRIN2A
KEAP1
AKT1
NFE2L2
FLNA
EGFR
Number of events
SNA-based tumor suppressor events
TP53
ARID1A
APC
KMT2D
ATM
CDKN2A
MLL2
NCOR1
ARHGAP35
KMT2C
Number of events
CNA-based tumor suppressor events
RB1
SMAD4
CDKN2A
ARID1A
PTEN
EP300
PIK3R1
FBXW7
SMAD2
NF1
Number of events
Mixed tumor suppressor events
TP53
APC
RB1
PTEN
STK11
NF1
EP300
SMAD4
RASA1
RBL2
Number of events
Driver chromosome losses
13
21
4
15
22
18
14
9
10
11
Number of events
Driver chromosome gains
20
7
8
13
12
5
2
16
14
1
Number of events
Driver arm losses
8p
17p
5q
3p
1p
16q
6q
19p
9p
18q
Number of events
Driver arm gains
5p
8q
1q
3q
6p
20q
16p
7p
2p
17q
Number of events
Driver events of all classes
TP53
8p
17p
13
5q
PIK3CA
21
5p
8q
1q
Number of events
